# Development of a prognostic prediction model for non-smoking lung adenocarcinoma based on pathological information and laboratory hematologic indicators: a multicenter study

**DOI:** 10.3389/fimmu.2025.1566195

**Published:** 2025-03-14

**Authors:** Run Xiang, Peihong Hu, Xiaoxiong Xiao, Wen Li, Xiaoqing Liao, Jun Li, Wen Zhu, Xiaoqin Liu, Qiang Li

**Affiliations:** ^1^ State Key Laboratory of Biotherapy, Sichuan University, Chengdu, China; ^2^ Department of Thoracic Surgery, Sichuan Clinical Research Center for Cancer, Sichuan Cancer Hospital and Institute, Sichuan Cancer Center, University of Electronic Science and Technology of China, Chengdu, China; ^3^ Department of Thoracic Surgery, Xiangya Hospital, Central South University, Changsha, Hunan, China; ^4^ Xiangya Lung Cancer Center, Xiangya Hospital, Central South University, National Clinical Research Center for Geriatric Disorders, Changsha, China; ^5^ Department of Medical Oncology, Cancer Center, West China Hospital, Sichuan University, Chengdu, Sichuan, China; ^6^ Lung Cancer Center, West China Hospital, Sichuan University, Chengdu, Sichuan, China; ^7^ Department of Thoracic Surgery, Dazhu County People’s Hospital, Dazhou, Sichuan, China; ^8^ Department of Thoracic Surgery, Ziyang Yanjiang People’s Hospital, Ziyang, Sichuan, China

**Keywords:** non-smoking lung adenocarcinoma, proportional hazards model, general pathological information, nomogram, prognostic prediction

## Abstract

**Objective:**

To develop a simple and practical model to predict the prognostic survival of non-smoking patients with lung adenocarcinoma by combining general pathological information with laboratory hematologic indicators.

**Methods:**

Cox univariate and multivariate analyses were used to identify the variable indicators. A Cox proportional hazards model was constructed based on the selected variables to compare survival outcomes between the high-and low-risk groups of non-smoking patients with lung adenocarcinoma and to validate the model’s performance. Subsequently, a nomogram model was established to systematically evaluate the impact of selected variables on prognosis.

**Results:**

Data of non-smoking patients with lung adenocarcinoma from four hospitals were retrospectively collected. We enrolled 1,172 patients, this includes 372 external validation data. Multivariate analysis identified six significant variables (*P* < 0.05): tumor TNM stage, tumor size, white blood cell count, neutrophil percentage, lymphocyte percentage, and hemoglobin level. We combined these six variables to build a model. The C-index of the training set is 0.811 (0.780–0.842), this value is 0.786 (0.737–0.835) in,test set and 0.810 (0.772–0.847) in validation set. The area under the curve (AUC) results of the predicted 3-years overall survival (OS) of the three data sets were 0.850, 0.819, and 0.860, respectively. These values for 5-years were 0.811, 0.771, and 0.849. Stratified analysis based on tumor staging showed that the model effectively distinguished outcomes (*P* < 0.0001). High-risk groups demonstrated significantly poorer prognosis compared to low-risk groups (*P* < 0.001).

**Conclusion:**

The prognostic model based on tumor TNM stage, tumor size, white blood cell count, neutrophil percentage, lymphocyte percentage, and hemoglobin levels effectively predicted the prognosis of non-smoking patients with lung adenocarcinoma. Compared with the more studied blood markers at present, the indicators of our model do not need conversion, Our model provides a useful reference for personalized diagnosis and treatment in clinical practice.

## 1 Introduction

The 2022 global cancer statistics report revealed that among 36 types of cancer across 185 countries, Lung cancer has the highest incidence, at the same time, lung cancer also leads the death rate (1). Lung adenocarcinoma is the most studied type of lung cancer due to its high incidence rate in all subtypes of lung cancer. A 2023 article published in *The Lancet Oncology* on the global distribution of lung cancer subtypes reported that adenocarcinoma constitutes 39% of lung cancers among men and 57% among women, compared to 25% and 12%, respectively, for squamous cell carcinoma (2). Smoking is a well-established independent risk factor for lung cancer. By 2024, it is estimated that 81% of lung cancer deaths will be directly attributable to smoking (5). In some countries, a decrease in the prevalence of smoking has been accompanied by a decline in lung cancer incidence (3). However, approximately 25% of lung cancer cases globally are attributed to factors other than smoking (4). Early diagnosis and treatment strategies for non-smoking patients with lung cancer may differ slightly from those for smokers. Therefore, early prediction of prognosis in non-smoking patients with lung cancer is critical for designing personalized treatment plans. Previous studies have rarely distinguished patients with lung cancer according to their smoking status to compare research outcomes.

Traditionally, laboratory test data have been an essential tool for supporting clinical diagnosis and evaluating treatment efficacy. In recent years, with the rise of bioinformatics, clinical laboratory omics have been recognized for their potential to uncover previously unexplored clinical value. For instance, platelets, long known for their critical role in thrombosis, have recently garnered attention as biomarkers for monitoring tumor progression (6, 7). Similarly, serum albumin has been identified as a biomarker for small-cell lung cancer patients undergoing immunotherapy (8). Additionally, the neutrophil-to-lymphocyte ratio (NLR) has shown potential as a prognostic marker in patients with colon cancer patients with isolated liver metastases (9). Therefore, developing a prognostic prediction model for lung cancer based on common blood biomarkers is a highly promising area of investigation.

This study was intended to offer valuable perspectives for clinical decision-making and explore the potential of blood biomarkers for predicting prognosis in non-smoking lung adenocarcinoma patients. The indicators used in this study, including neutrophil percentage, lymphocyte percentage, hemoglobin level, and white blood cell count, are routinely available from complete blood count (CBC) tests. These metrics can be directly measured without additional calculations, making them the most accessible and fundamental clinical testing parameters. Their low cost, simplicity, and feasibility make them practical tools for predicting lung cancer ending.

## 2 Methods

### 2.1 Patients selection

**Figure 1:**
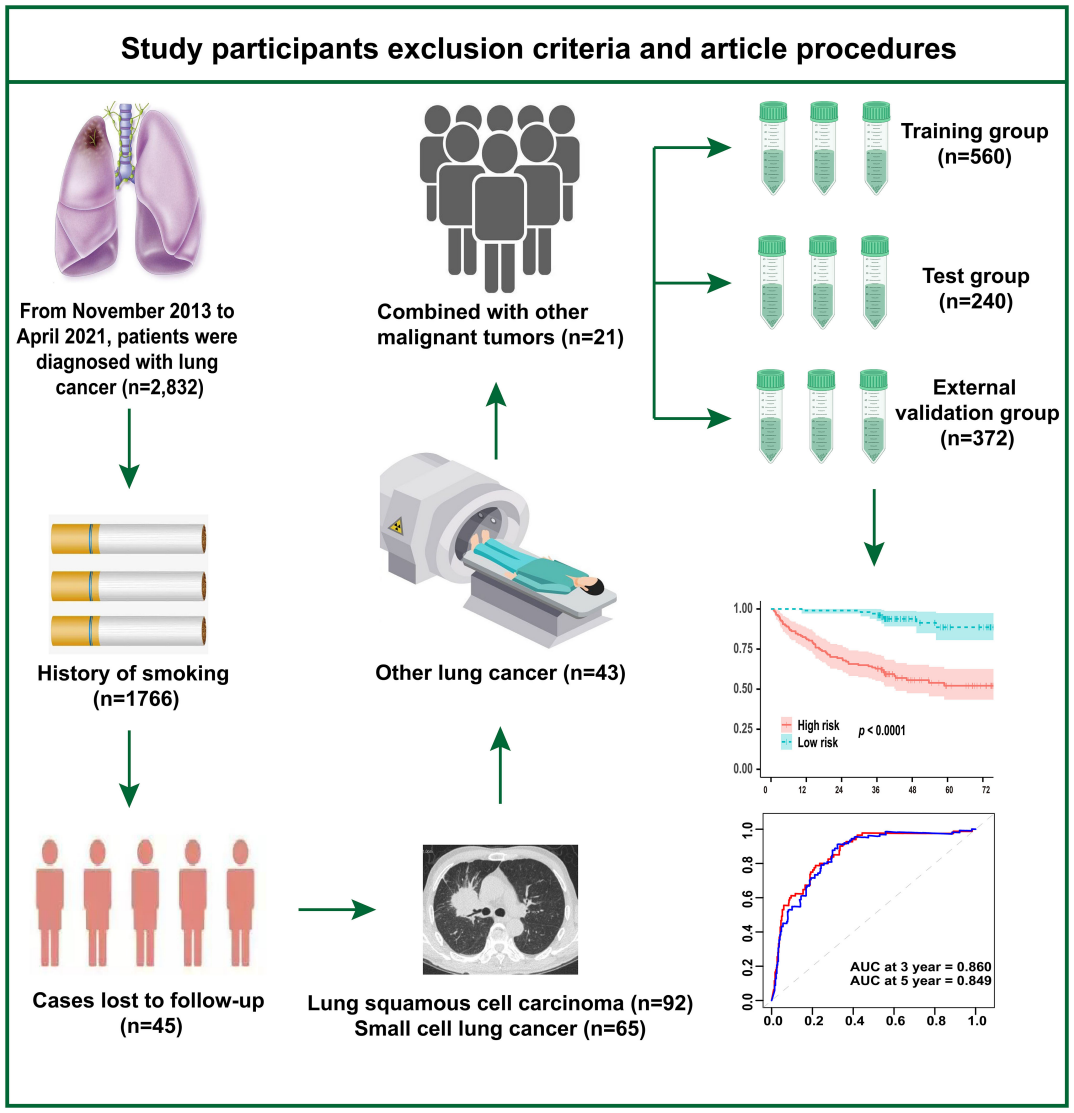
Study participants’ exclusion criteria and article procedures.

**Figure 1** illustrates the study’s inclusion and exclusion criteria, as well as the study workflow. We retrospectively analyzed 2,832 cases of lung cancer diagnosed at Sichuan Cancer Hospital between November 2013 and April 2021. The ones that followed were the exclusion criteria: (1) individuals who have smoked in the past; (2) individuals with small cell carcinoma, squamous cell carcinoma, or other forms of lung cancer; (3) individuals with other concurrent malignancies; and (4) individuals lost to follow-up. Additionally, using the same inclusion and exclusion criteria, we collected samples from four hospitals—West China Hospital of Sichuan University, Xiangya Hospital of Central South University, Dazhu County People’s Hospital, and Yanjiang District People’s Hospital of Ziyang—for external validation of the model. The Sichuan Cancer Hospital’s Ethics Committee gave its approval to this study (No. KY-2021-076). The informed consent exemption statement was carried out, and our investigation was retrospective.

### 2.2 Study design

The end point of this study was overall survival (OS). All included patients were followed up via telephone, and general patient information, such as sex, age, smoking history, tumor size, and tumor stage, was extracted from hospital integrated information system. The clinical blood test data used in the study were obtained from CBC results, which were analyzed using an automated hematology analyzer (Shenzhen Mindray, BC-5390).

### 2.3 Variable screening

Cox univariate analysis was performed to screen the CBC results of the included cases. Variables with *P* < 0.05 in the univariate analysis were subsequently subjected to Cox multivariate analysis to identify the key hematologic biomarkers for the study.

### 2.4 Model construction and evaluation

A training set and a test set were randomly selected from the modeling group data in a 7:3 ratio. The prediction model was established using the training set, and the model was internally verified using the test set, and the validation set data were used for external verification of the prediction performance of the model. Harrell’s C-index was the main evaluation index of the model. Additionally, the Bootstrap self-sampling method was used with 500 sampling times. A time-dependent receiver operating characteristic (ROC) curve with the area under the curve (AUC) was used to evaluate the prediction performance. Additionally, we create the model’s ROC curves for patients at 3- and 5- years, and we use the AUC to judge the model’s accuracy. The calibration curve and decision curve were used to evaluate the calibration performance and clinical applicability of the model, respectively. According to the median OS prediction results of the model for the patients, those in the training, test, and validation sets were divided into high- and low-risk groups. Participants were split into different risk(high and low) groups for the training, test, and validation sets. The survival curves were created using the Kaplan–Meier method, and the prognostic results of each risk grouping were compared using log-rank test. Finally, we used a nomogram to visualize the established model.

### 2.5 Statistical methods

To conduct statistical studies, R software (version 4.1.0) was utilized. Continuous variables were tested for normality, when describing the ones who did not fit into a normal distribution, the median (25th to 75th percentiles; M [P25, P75]) was selected. The Kruskal–Wallis test was used for comparisons of continuous variables, whereas the chi-squared test was applied for categorical variables. Statistical significance was set at *P* < 0.05.

## 3 Results

### 3.1 Clinical pathological characteristics of non-smoking patients with lung adenocarcinoma

800 patients who did not smoke in total with lung adenocarcinoma diagnosed at Sichuan Cancer Hospital were included in this study. Additionally, 372 patients from four other hospitals—West China Hospital of Sichuan University, Xiangya Hospital of Central South University, Dazhu County People’s Hospital, and Yanjiang District People’s Hospital of Ziyang—were used as the external validation cohort. Lung cancer pathology staging was determined based on the eighth edition of the National Comprehensive Cancer Network (NCCN) lung cancer classification standards. All of the patients’ median ages were 60 (56.4, 70.4) years. The median duration of follow-up was 42.3 (36.5, 59.7) months. Among all patients, 902 (77.0%) were female, and 322 (27.5%) died. Among all patients, 233 (19.9%) were classified as Stage III and 253 (21.6%) as Stage IV. The basic data of the patients in the training, test, and validation sets are shown in **Table 1**.

**Table 1:** Clinical features of 1172 patients with LUAD.

Variables	Training set (n = 560)	Test set (n = 240)	Validation set (n = 372)	Statistic	*P*
Time, Month	40.7 (36.3,58.5)	40.0 (36.5,57.8)	54.0 (40.3,64.5)	χ² = 27.91^#^	<.001
Size, mm	20 (17,32)	20 (17,33)	24 (16,36)	χ² = 2.88^#^	0.237
Age, year	60 (51,66)	58 (50,67)	62 (55,68)	χ² = 20.05^#^	<.001
WBC	5.58 (4.62,6.77)	5.65 (4.62,6.92)	5.67 (4.60,7.11)	χ² = 0.60^#^	0.741
Percentage of Neutrophil	64.3 (57.0,70.7)	63.8 (55.8,70.0)	64.0 (56.4,69.7)	χ² = 0.08^#^	0.960
Percentage of Lymphocyte	26.6 (20.4,33.4)	26.4 (21.2,33.9)	21.7 (1.7,31.9)	χ² = 62.79^#^	<.001
Percentage of Monocyte	5.8 (4.8,6.7)	6.0 (4.8,6.9)	6.3 (5.1,7.7)	χ² = 22.51^#^	<.001
Hb	130 (120,139)	129 (120,137)	127 (118,137)	χ² = 4.36^#^	0.113
Status, n (%)				χ² = 0.15	0.926
Alive	406 (72.5)	172 (71.7)	272 (73.1)		
Dead	154 (27.5)	68 (28.3)	100 (26.9)		
Sex, n (%)				χ² = 7.26	0.026
Female	450 (80.4)	180 (75.0)	272 (73.1)		
Male	110 (19.6)	60 (25.0)	100 (26.9)		
Stage, n (%)				χ² = 12.23	0.057
I	277 (49.5)	103 (42.9)	208 (55.9)		
II	42 (7.50)	24 (10.0)	32 (8.6)		
III	112 (20.0)	57 (23.8)	64 (17.2)		
IV	129 (23.0)	56 (23.3)	68 (18.3)		

^#^Kruskal–Wallis test, χ²Chi-squared test.

### 3.2 Univariate and multivariate Cox analysis

**Figure 2:**
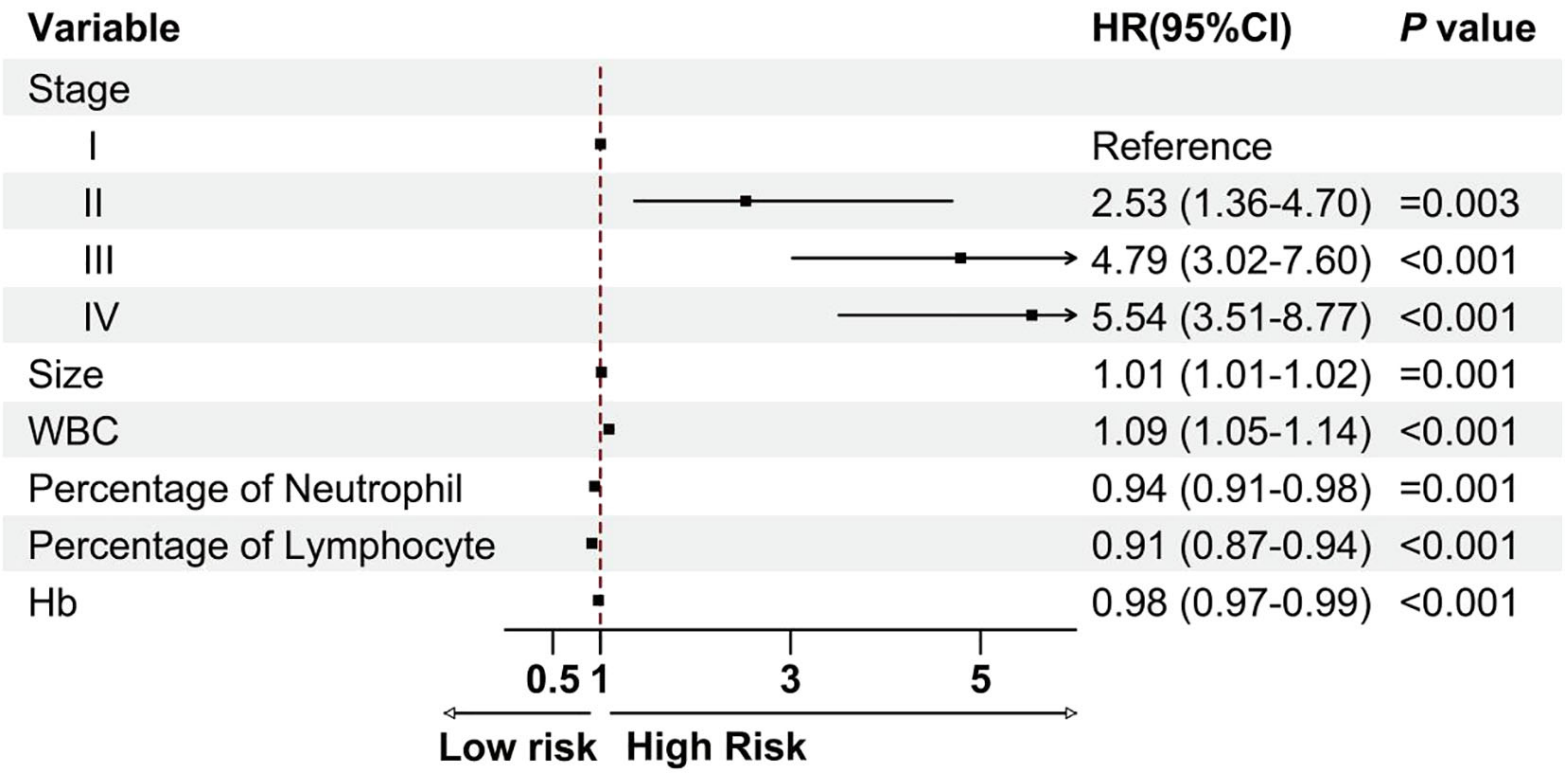
Cox multivariate analysis was used to screen clinical research variables.

We carried out univariate and multivariate Cox analyses to investigate the association between the clinical features and OS. The results identified the following independent prognostic factors for patient outcomes: tumor TNM stage, tumor size (hazard ratio (HR): 1.01, 95% confidence interval (CI):1.01–1.02, *P*= 0.001), white blood cell count (HR: 1.09, 95% CI: 1.05–1.14, *P*< 0.001), neutrophil percentage (HR: 0.94, 95% CI: 0.91–0.98, *P*= 0.001), lymphocyte percentage (HR: 0.91, 95% CI: 0.87–0.94, *P*< 0.001), and hemoglobin level (HR: 0.98, 95% CI: 0.97–0.99, *P<* 0.001; **Figure 2**). These four laboratory markers (white blood cell count, neutrophil percentage, lymphocyte percentage, and hemoglobin level) significantly affect patient prognosis.

### 3.3 A six-variable combined model for predicting prognosis in non-smoking patients with lung adenocarcinoma

**Figure 3:**
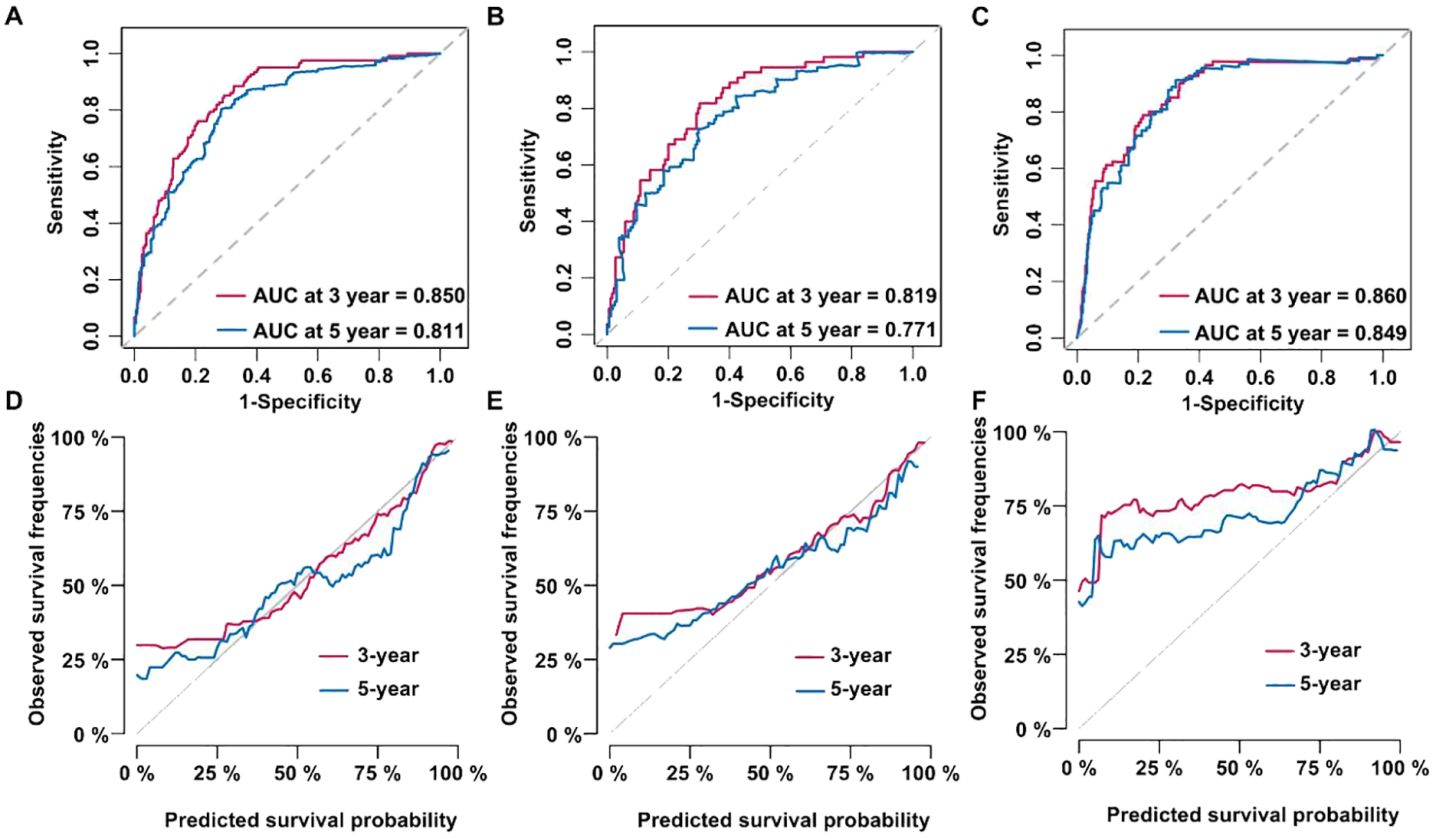
Cox proportional hazard models were established and validated. **(A)** Receiver operating characteristic (ROC) curve of the training dataset. **(B)** ROC curve of the test dataset. **(C)** ROC curve of the validation dataset. **(D)** Calibration curve of the training dataset. **(E)** Calibration curve of the test dataset. **(F)** Calibration curve of the validation dataset.

A model of Cox proportional hazards was established with the variables chosen from the Cox multivariate analysis. In the training cohort, the ROC curve analysis revealed that the AUC for the 3- and 5-years OS was 0.850 and 0.811 (**Figure 3A**). In the test cohort, the AUC was 0.819 and 0.771, respectively (**Figure 3B**). Finally, the AUC in the external validation cohort was 0.860 and 0.849, respectively (**Figure 3C**). **Figures 3D–F** show the calibration curves for the training, testing, and validation cohorts.

**Figure 4:**
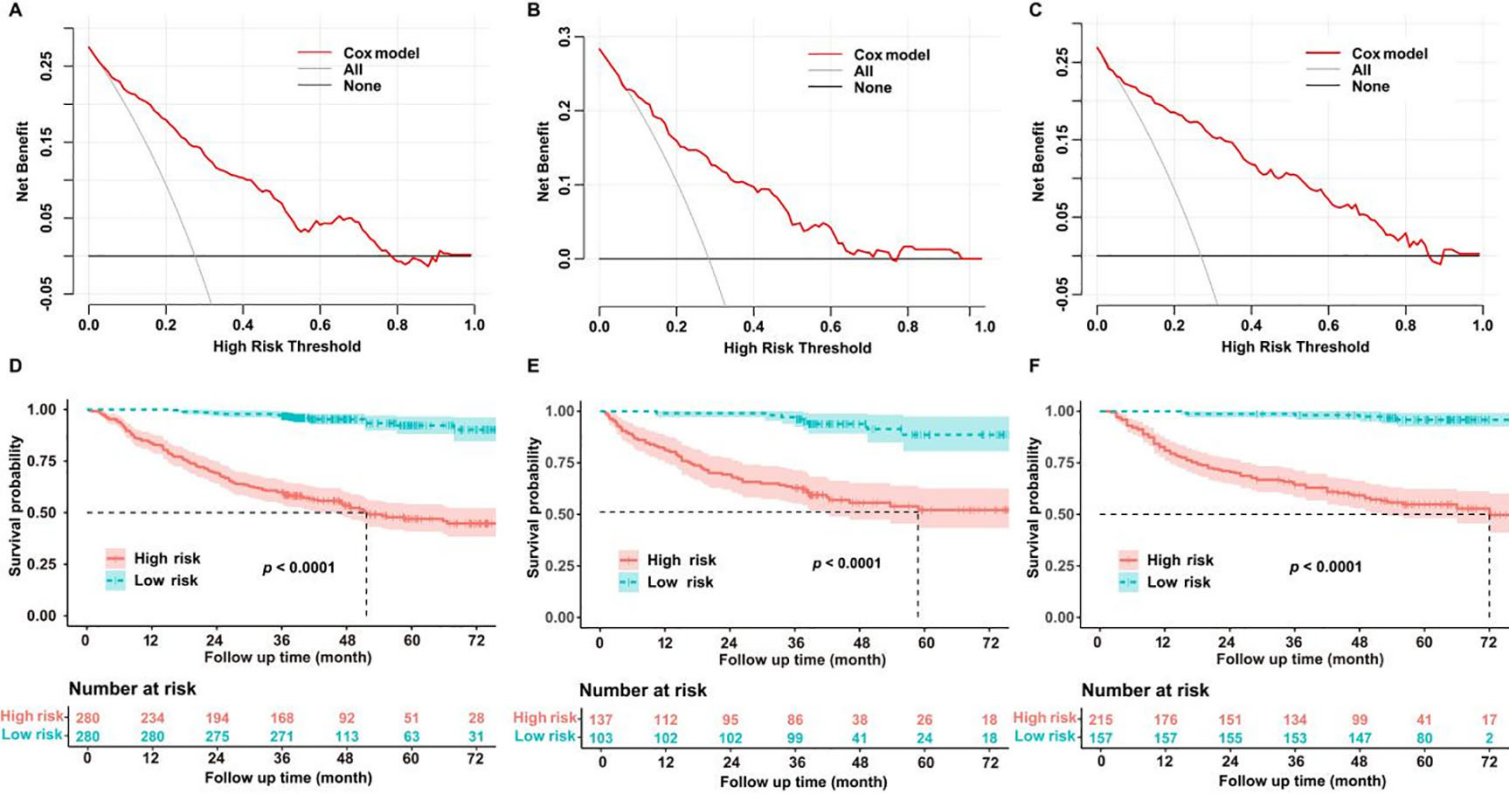
**(A)** Decision curve analysis (DCA) curve of the training dataset. **(B)** DCA curve of the test dataset. **(C)** DCA curve of the validation dataset. **(D)** Kaplan–Meier curve of the training dataset. **(E)** Kaplan–Meier curve of the test dataset. **(F)** Kaplan–Meier curve of the validation dataset.

Decision curve analysis (DCA) was used to assess the clinical benefits of our model. Within the training dataset, the threshold probability with a practical value ranged from 0 to 0.75 (**Figure 4A**); in the testing cohort, it ranged from 0 to 0.7 (**Figure 4B**); and in the validation cohort, it ranged from 0 to 0.85 (**Figure 4C**). Patients were separated into different risk groups (high or low) using the median values of tumor TNM stage, tumor size, white blood cell count, neutrophil percentage, lymphocyte percentage, and hemoglobin level as cutoff points. Kaplan–Meier analysis was performed to evaluate the relationship between these variables and OS in non-smoking patients with lung adenocarcinoma. Survival analysis demonstrated remarkable dissimilarities in OS between the high- and low-risk sets in the training (**Figure 4D**), testing (**Figure 4E**), and validation datasets (**Figure 4F**; *P* < 0.0001).

**Figure 5:**
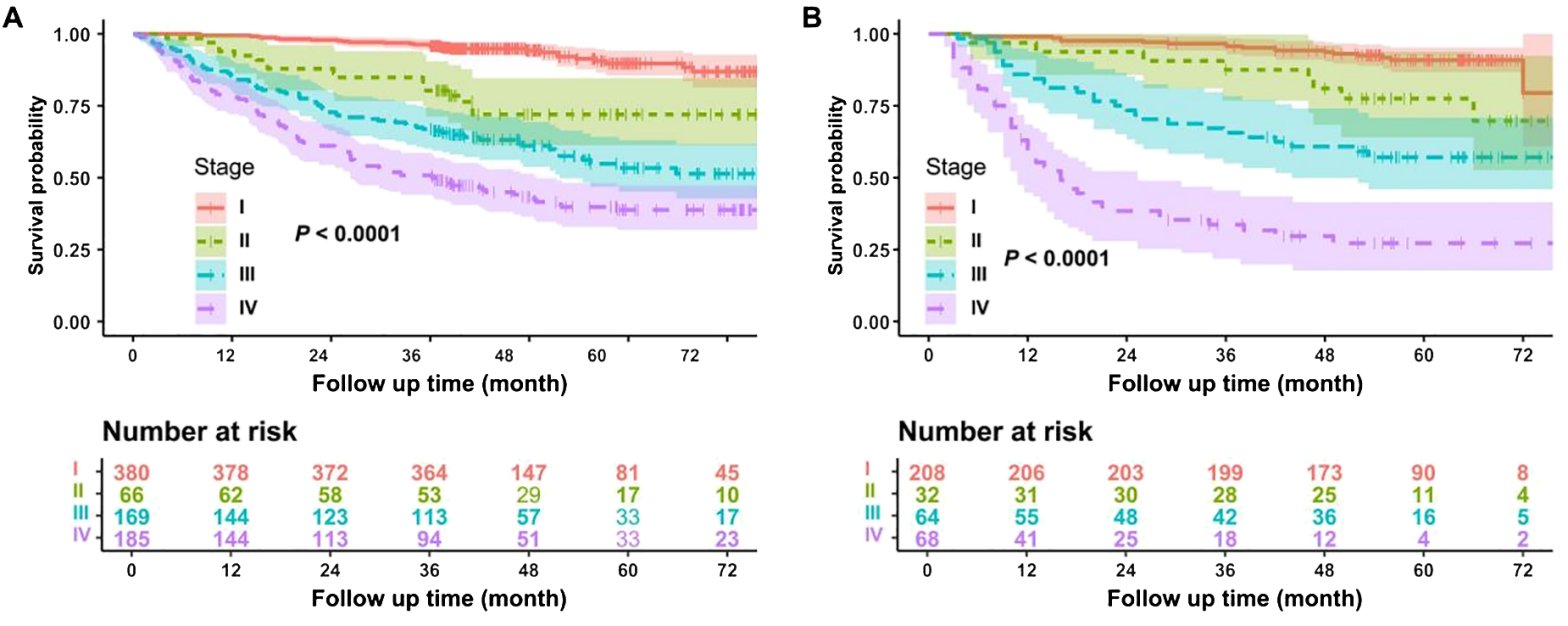
Subgroup analysis of the model. **(A)** Subgroup analysis of the modeling dataset. **(B)** Subgroup analysis of the validation dataset.

The model’s predictive ability across different clinical stages of non-smoking lung adenocarcinoma was a key focus of this study. By using the log-rank test method, **Figure 5A** shows the subgroup analysis results for different pathological stages in the training cohort, whereas **Figure 5B** presents the subgroup analysis results in the validation cohort. In both the modeling and validation datasets, the survival curves between different stages of lung adenocarcinoma were different, and the difference was statistically significant: the survival of stage I and II was better than that of stage III and IV, *P* < 0.0001.

### 3.4 Establishment and validation of the nomogram based prognostic prediction model

**Figure 6:**
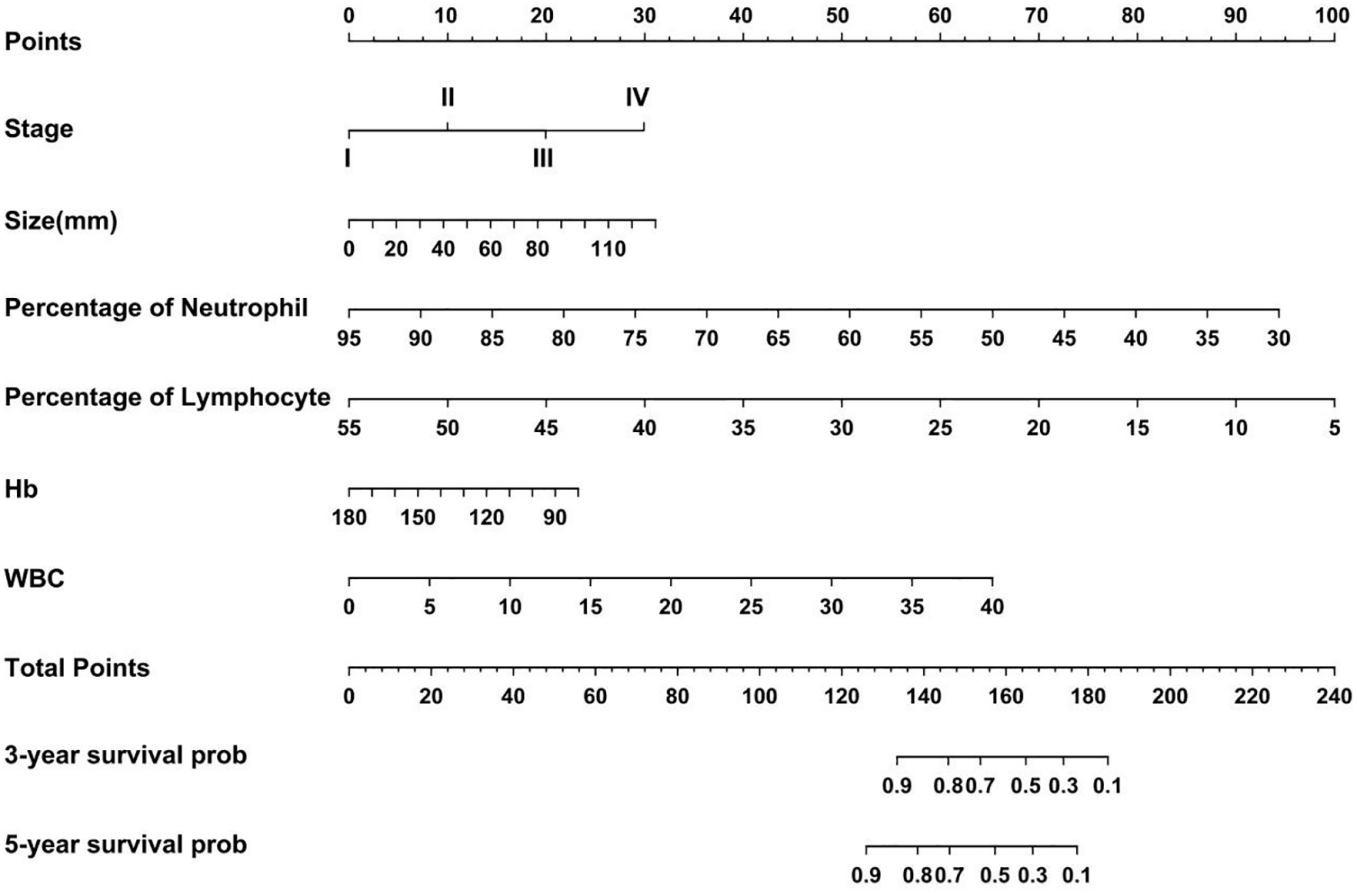
Nomogram for predicting the prognosis of lung adenocarcinoma in non-smoking patients.

Based on the regression coefficients of the influencing factors from the Cox proportional hazards model, scores were assigned to each of the following factors: pathological stage, tumor size, white blood cell count, neutrophil percentage, lymphocyte percentage, and hemoglobin level. The scores for each factor were summed to obtain a total score. This total score was converted into an estimated survival rate, which was then used to calculate the predicted tumor progression value and create a nomogram for predicting the prognosis of non-smoking patients with lung adenocarcinoma (**Figure 6**). Each factor was represented by a line segment on the nomogram, with scale marks indicating the range of possible values for that factor. The length of the line segment reflects the size of the regression coefficient of the factor in relation to the outcome. The total score, including both individual and cumulative scores, was determined by drawing a vertical line to determine the corresponding 3- and 5-years survival rates. Bootstrap resampling was performed 500 times to validate the proposed model. The C-index values for the training, testing, and validation cohorts were greater than 0.7 (**Supplementary Figure S1A–C**), indicating that the model had significant predictive power.

## 4 Discussion

In recent years, research on prognostic biomarkers of lung cancer has gained significant attention; however, most studies have been limited to data from approximately 200 patients. In our retrospective analysis, we used data from nearly 1,200 cases to construct a prognostic prediction model for non-smoking lung adenocarcinoma based on blood analysis indicators. Furthermore, we conducted a multicenter external validation to assess the prognostic efficacy of peripheral blood markers. Our results demonstrated that the high-risk group, characterized by later tumor stages, larger tumor size, higher white blood cell count, neutrophil percentage, lymphocyte percentage, and hemoglobin levels, had a markedly worse prognosis than the low-risk group. Additionally, we developed a nomogram for prognostic prediction that serves as a valuable reference for assessing the prognosis of these patients.

White blood cells comprise neutrophils, lymphocytes, monocytes, and other types. The roles of different white blood cell components have been widely explored. Generally, white blood cells represent the body’s level of inflammation, which has a critical function in the development and evolution of tumors (10–12). Some scholars have suggested that chronic inflammation is a constant feature during tumor development, leading to the idea that tumors are “non-healing wounds” (13). In previous studies, the NLR has been frequently used as a prognostic marker. The NLR has different predictive roles in various types of cancer (14–16); however, its value as a prognostic predictor remains controversial.

Some studies have suggested that the lymphocyte percentage may better reflect cancer prognosis than the absolute lymphocyte count (17). Researchers have indicated that a low lymphocyte percentage could serve as a marker for poor prognosis (18, 19), whereas others have found that high neutrophil and lymphocyte percentages are linked to a worse prognosis in patients suffering from colorectal cancer (20). Research specifically focusing on the lymphocyte percentage in lung cancer is limited. In this study, we incorporated both neutrophil and lymphocyte percentages into our model. These two markers are directly available in routine blood tests without the need to calculate a ratio, making them more feasible for clinical application.

Hemoglobin, which is primarily found in red blood cells, is closely associated with tumor development. Existing research suggests that low hemoglobin levels lead to tissue hypoxia, triggering a series of body responses (21, 22). A retrospective analysis based on a European population showed that high hemoglobin levels were beneficial in reducing the incidence of prostate cancer (23, 24); however, in other malignancies, hemoglobin levels may exhibit different effects. For example, high hemoglobin levels may augment the danger of cervical cancer and melanoma (25).

The influence of tumor staging and tumor size on prognosis is straightforward. Later-stage tumors generally have a poorer prognosis, and larger tumor diameters are often associated with more complications, leading to worse outcomes. On the other hand, blood test indicators have varying effects across different cancer types. Additionally, even within the same cancer type, the effects can differ at various stages or across different patient populations, possibly due to the inherent heterogeneity of tumors. Therefore, our study did not focus on a single marker but instead combined several risk factors into a model to explore their role in predicting the prognosis of non-smoking lung adenocarcinoma. Moreover, in clinical practice, these indicators are simple, readily available, and do not require complex calculations, which enhances their clinical applicability. This is a key advantage of this study. By providing individualized risk predictions and dynamic updates, our model offers actionable insights that can directly inform clinical decision-making, a feature that is often lacking in traditional risk scoring systems. In terms of innovation, the model performs stably in the external validation set, avoiding the overfitting problem that may exist in traditional methods. In addition, our model can more finely stratifying or predicting the risk of individual patients, thus providing support for personalized treatment.

Our study has some limitations. There are inevitable biases in sample selection, and our prediction model may perform well in specific regions and specific populations, but there may be insufficient generalization ability in different medical institutions or populations, which needs to be verified and optimized in large sample environment. In reality, the outcome of the disease may be improved by treatment, therefore, in future work, we will also incorporate propensity-score matching or difference-in-differences methods into our models to assess treatment effects on disease outcomes.

## 5 Conclusion

In conclusion, this study aimed to analyze the prognosis of non-smoking patients with lung adenocarcinoma using a combined model including tumor stage, tumor size, white blood cell count, neutrophil percentage, lymphocyte percentage, and hemoglobin level. The nomogram developed in our research can effectively predict the prognosis of non-smoking patients with lung adenocarcinoma, helping clinicians screen high-risk patients and offering personalized treatment.

## Data Availability

The original contributions presented in the study are included in the article/**Supplementary Material**. Further inquiries can be directed to the corresponding authors.

## Supplementary material

Supplementary Figure 1 Self-sampling validation of the model. **(A)** Self-sampling validation of the training dataset. **(B)** Self-sampling validation of the test dataset. **(C)** Self-sampling validation of the validation dataset.
