# Supplementary figures and images for: Development of a prognostic prediction model for non-smoking lung adenocarcinoma based on pathological information and laboratory hematologic indicators: a multicenter study
Source: Front Immunol. 2025 Mar 14;16:1566195. doi: 10.3389/fimmu.2025.1566195 (PMC11949898; doi:10.3389/fimmu.2025.1566195)

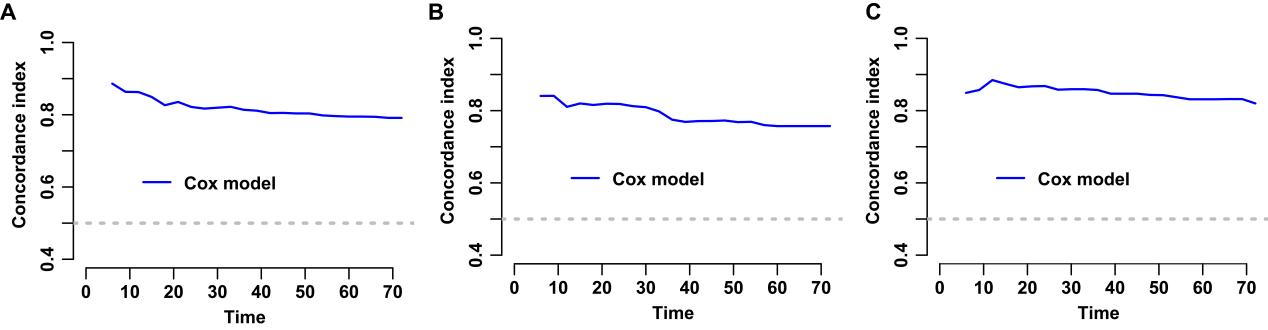

Supplement: Supplementary Figure 1 — Self-sampling validation of the model. (A) Self-sampling validation of the training dataset. (B) Self-sampling validation of the test dataset. (C) Self-sampling validation of the validation dataset. [file Image1.tif]
